# Congenitally Corrected Transposition of Great Arteries with Dextrocardia, Patent Ductus Arteriosus, Atrial Septal Defects and Ventricular Septal Defects in a 15-Year-Old Marfanoid Habitus Patient: A Case Study

**DOI:** 10.7759/cureus.8937

**Published:** 2020-06-30

**Authors:** Misbah Munaf, Sofia Farooqui, Syeda K Kazmi, Ibtehaj Ul-Haque

**Affiliations:** 1 Internal Medicine, Dow University of Health Sciences, Karachi, PAK

**Keywords:** dextrocardia, patent ductus arteriosus, atrial septal defect, marfanoid habitus, pulmonary atresia, ventricular septal defect, congenitally corrected transposition of the great arteries

## Abstract

Congenitally corrected transposition of the great arteries (CCTGA) is a rare congenital cardiac anomaly defined by atrio-ventricular and ventriculo-arterial discordance. This malformation makes up less than 1% of congenital heart defects. We report here a case of a 15-year-old female who presented to our hospital with dyspnea as seen in the New York Heart Association (NYHA) Functional Classification class III and hemoptysis. She was clinically found to have marfanoid habitus, and subsequent echocardiographic study disclosed CCTGA-associated with Ebstein's anomaly, ventricular septal defect, left ventricular outflow obstruction, right ventricular outflow obstruction, co-existing dextrocardia, atrial septal defect, patent ductus arteriosus, non-confluent pulmonary arteries, and pulmonary atresia. This case highlights the association between such rare cardiac conditions. To the best of our knowledge, this is the first case of CCTGA at a young age, with the aforementioned abnormalities documented in the literature reported from Pakistan.

## Introduction

Congenitally corrected transposition of the great arteries (CCTGA) is a rare cardiac condition with atrio-ventricular and ventriculo-arterial discordance (transposition of the great arteries), representing less than 1% of all congenital cardiac diseases [1-3]. Also commonly referred to as levo or L-looped transposition of the great arteries (L-TGA), double discordance, or ventricular inversion.

The normal looping of the heart occurs in the third week of development. Normally the primitive heart loops to the right, resulting in the placement of right ventricle (RV) to the right of the left ventricle (LV). If the looping occurs to the left, the RV is placed on the left with LV on the right, causing ventricular discordance. This results in CCTGA and changes the route of the blood flow. The venous return is to the right atrium (RA), from where blood is conducted via the mitral valve to the LV. Then the pulmonary artery (PA) takes the blood to the lungs and the pulmonary vein (PV) returns it to the left atrium (LA). From there the blood travels via the tricuspid valve to the RV and finally via the aorta to the systemic circulation. So in isolated CCTGA, although the anatomy is disturbed, there is no mixing of deoxygenated and oxygenated blood and the patient is asymptomatic at birth and through childhood. Later in the disease, pressure overload can cause RV dysfunction, due to its unfavorable tripartite geometric configuration [4].

Associated cardiac lesions are present in over 90% of patients with L-TGA. The associated anomalies include patent ductus arteriosus, secundum atrial septal defect, large inlet ventricular septal defect, and pulmonary atresia.

Another variant of CCTGA is dextrocardia; the anatomy is different but the pathophysiology is the same. In dextrocardia, the heart deviates to the right side of the hemithorax from its normal left position. When dextrocardia with situs solitus occurs, the morphological ventricles are inverted however the atria and the other abdominal viscera are present in their normal anatomical positions [5-6].

The most common associations with CCTGA, seen in 80% of all cases, are ventricular septal defect (VSD), pulmonary stenosis (PS), left atrioventricular (AV) valve (morphological tricuspid valve) regurgitation and/or complete heart block [2-3, 7]. 

## Case presentation

We present a case of a 15-year-old Pakistani female admitted from our outpatient department (OPD) to the cardiology ward with shortness of breath and worsening hemoptysis for the preceding two days. A similar episode of hemoptysis took place one year prior, which resolved spontaneously on the administration of an unknown drug. No investigation was done at the time. At the age of 12 years she was diagnosed with a congenital cardiac anomaly, tetralogy of Fallot (TOF), and was managed conservatively without any surgical procedures. She was admitted to our hospital for further evaluation. The patient had a history of recurrent cyanosis of fingers, toes, and tongue since birth. Initially, she had dyspnea consistent with New York Heart Association (NYHA) Functional Classification class II (light limitation of physical activity with ordinary physical activity resulting in fatigue, palpitation, dyspnea) which progressed to class III in the past four years (marked limitation of physical activity with less than ordinary activity causing fatigue, palpitation or dyspnea).​​​​There were no previous reports of palpitation, chest pain, or pedal edema. Her family history was insignificant for cardiac diseases.

On physical examination, the subject appeared cachexic with signs of clubbing. She was cyanosed with a high arched palate, arm span that exceeded her height, thin long slender fingers with pes cavus, and a positive thumb sign indicating the condition of marfanoid habitus. The hemodynamic values included a regular pulse rate of 100 beats/minute and blood pressure of 100/70 mmHg. Respiratory vitals were varied but stayed within normal range showing 80% transcutaneous oxygen saturation with a respiratory rate of 24 bpm. Auscultation of the patient revealed a loud single second heart sound (S2) at the left second intercostal space. A grade 3/6 systolic murmur was audible along the lower right sternal border that increased with inspiration. Her cardiac apex impulse was palpated in the fifth intercostal space 1 cm medial to mid-clavicular line on the right side of her chest.

**Figure 1:**
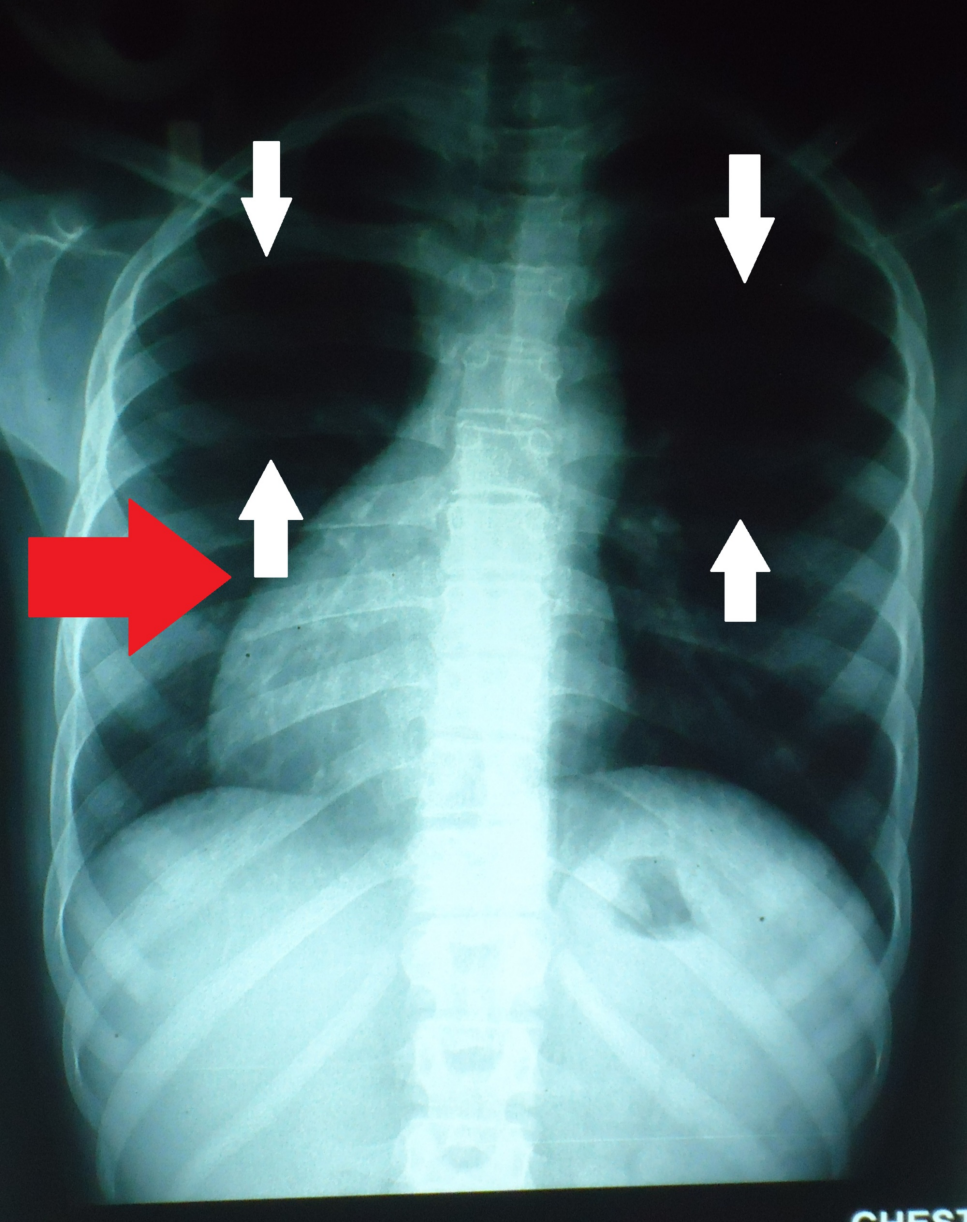
Posteroanterior chest X-ray PA showing oligemic lung fields (see white arrows). The red arrow indicates dextrocardia

Figure 1 shows a chest radiograph of posteroanterior view with decreased right lung volume. The heart is also seen to be displaced to the right side as pointed by the red arrow.

Transthoracic echocardiography (TTE) was performed for optimal evaluation (Video 1) which revealed a double atrioventricular and ventriculo-arterial discordance. The morphology of the right ventricle was noticed by the presence of coarse trabeculations and a moderator band with the mitral valve apically displaced to the tricuspid valve. Assessment of the outflow tract suggested the aorta originating from the morphological right ventricle, being anterior and to the left of the pulmonary artery. The right A-V valve had mild to moderate regurgitation. TTE also showed an ostium secundum atrial septal defect (ASD) measuring 10 mm in diameter with bi-directional shunting flow through the defect. A 20 mm large inlet type VSD having a two-way shunt was observed. There was also evidence of pulmonary atresia and a 4 mm in diameter patent ductus arteriosus (PDA) with the left to right flow, a possible connection between the left main pulmonary artery and thoracic aorta. Furthermore, the patient also had non-confluent pulmonary arteries. Rupture of these non-confluent pulmonary capillaries led to the presenting complaint of hemoptysis. The systemic ventricle (morphological right ventricle) function was nearly normal with an ejection fraction of 65%. No other concomitant disorders, such as aortic coarctation, ventricular dysfunction, and pericardial effusion diseases or thrombus were detected.

**Video 1:** Echocardiogram of the patient shows ostium secundum atrial septal defect and large inlet ventricular septal defect

**Figure 2:**
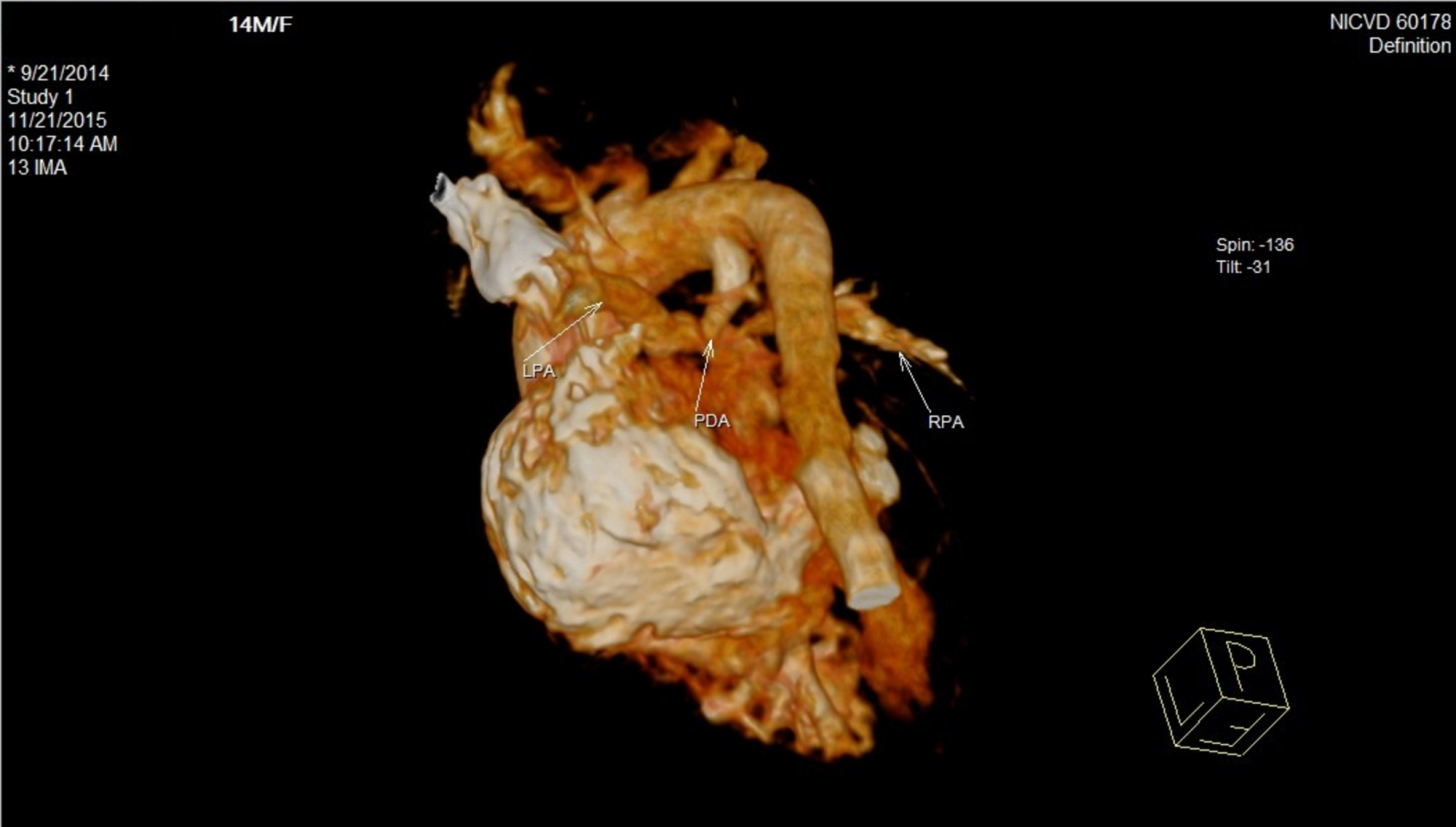
Cardiac CT scan showing patent ductus arteriosus in the patient RPA, right pulmonary artery; LPA, left pulmonary artery; PDA, patent ductus arteriosus

**Figure 3:**
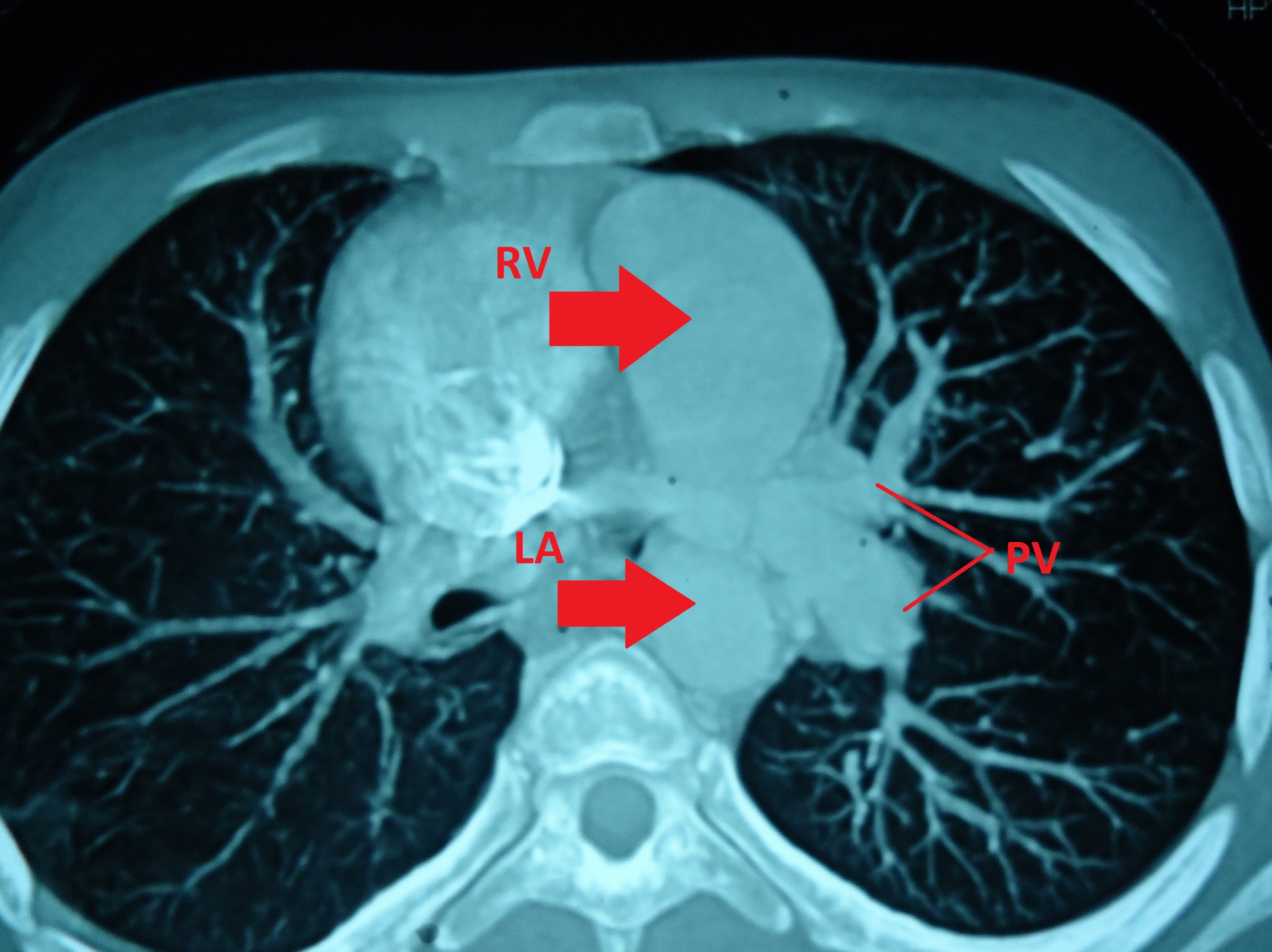
CT Scan of the patient showing discordant positioning of LA and RV PV, Pulmonary vein; LA, left atrium; RV, right ventricle

We also performed a chest computed tomography (CT) scan. Figure 2 confirms the double discordance demonstrating PDA (as shown by the white arrow) and it also shows locations of right pulmonary (RPA) and left pulmonary artery (LPA). As shown in Figure 3, the right ventricular outflow tract showed no communication with pulmonary artery. The hypoplastic right ventricle and right main pulmonary artery were also visualized.

## Discussion

This is the first case in our hospital and possibly a rarity in Pakistan, which cannot be confirmed due to a virtually non-existent medical database. Therefore, this is perhaps the first report in the country addressing CCTGA in a young patient. 

CCTGA is a very rare congenital anomaly observed with a prevalence of less than 1% of all congenital heart diseases and is often diagnosed after 18-20 years of age [1, 8-9]. However, our patient presented at an atypical age of 15 years. Clinical presentation, functional status, and long-term outcomes are largely determined by associated defects and complications including septal defects, tricuspid valve anomalies, pulmonary stenosis, pulmonary atresia, AV blocks, etc [1, 9]. Patients with simple or isolated CCTGA tend to live beyond 40 years of age, while the average mortality of those with associated defects have been reported to be around 38.5 years [1]. 

Due to the presence of VSD and pulmonary atresia, it can be argued that the case presentation resembles TOF, a congenital heart disease characterized by the presence of the following: an overriding aorta, pulmonary infundibular stenosis, right ventricular hypertrophy, and VSD [10]. It can also co-exist with other conditions of the heart like ASD, pulmonary atresia, and non-confluent pulmonary arteries [11].

In contrast to TOF, CCTGA is characterized by the presence of double atrio-ventricular and arterio-ventricular discordance. Unlike TOF, CCTGA does not present with an overriding aorta. In this case, the aorta is placed left and anterior to the pulmonary artery instead of the normal left and posterior [12]. In addition to the transposed ventricles and arteries, CCTGA can also present with pulmonary atresia and pulmonary stenosis. ASD and VSD have also been seen to arise in these patients and exist independently [13]. Table 1 compares the features of TOF and CCTGA with the findings of our presented case.

**Table 1:** Comparison of features of TOF and CCTGA with the presented case TOF: Tetralogy of fallot; CCTGA: congenitally corrected transposition of great arteries; VSD: ventricular septal defect; ASD: atrial septal defect

	TOF	CCTGA	Presented Case
VSD	Present	Not present	Present
Overriding aorta	Present	Not present	Not present
Pulmonary stenosis	Present	Can present with but not seen in classical cases	Not present
Right ventricular hypertrophy	Present	Not present	Not present (systemic ventricle/morphological right ventricle was normal)
Pulmonary atresia	Can present with but not seen in classical cases	Can present with but not seen in classical cases	Present
Non-confluent pulmonary arteries	Can present with but not seen in classical cases	Can present with but not seen in classical cases	Present
ASD	Not present (observed to co-exist in some cases)	Not present (observed to co-exist in some cases)	Present
Atrioventricular and ventriculo-arterial discordance	Not present	Present	Present

Hence we deduce that although it closely resembles TOF, our patient’s findings of double atrio-ventricular and arterio-ventricular discordance along with the absence of an overriding aorta are comparable to the features of CCTGA, hence, this is a peculiar case of CCTGA with ASD, VSD, pulmonary atresia, and non-confluent pulmonary arteries. 

Due to anomalous anatomy and hemodynamics, right ventricular dysfunction (RVD) and tricuspid insufficiency (TI) ensues. RVD is probably caused by the inability to sustain systematic circulation and abnormal coronary supply, which can potentially lead to congestive heart failure (CHF) [14]. The failing ventricle's connection with tricuspid regurgitation is much debated but studies have provided data regarding patients with both the conditions in coexistence [1, 9, 15]. RV dysfunction is always preceded by TI and it is a potential predictor of death in CCTGA patients, with the survival rate greater than 90% in patients without TI [9]. The risk factor of developing TI is highly indicated with tricuspid valve anomaly.

Other complications include rhythm disturbances, heart blocks, atrial and ventricular arrhythmias respectively. Rhythm disturbances and heart blocks are linked to the abnormal position of AV nodes and conducting bundles; their incidences increasing each year by a rate of 2% per year [1]. Atrial arrhythmias are highly prevalent and when CCTGA is not associated with any other abnormalities, they appear around the fifth decade of life, in contrast with complicated cases when they appear earlier around the second or third decade of life [1].

Chest X-rays and ECG are primary modalities of choice, providing enough data for further investigations. Chest X-rays reveal abnormal contours while ECG can show a false sign of lower myocardial infarct, which is actually due to inverted bundles. MRI, echocardiogram, and catheterization are the most accurate and definitive procedures which reveal the precise morphological nature of the anomaly and also help in assessing the degree of tricuspid valve regurgitation, ventricular function, and effects of various anomalies on hemodynamics. Despite extensive cardiac imaging, these procedures require experts as the diagnosis goes unnoticed in some patients, as was the case with this patient.

The treatment approach that can be opted for this case includes bidirectional Glenn procedure and hemi-Fontan, followed by Fontan completion. In the bidirectional Glenn procedure, the oxygen-poor blood from SVC is redirected to the lungs by disconnecting the SVC from the heart and redirecting it to the pulmonary arteries. In the Fontan completion, the blood is redirected from the IVC to the lungs correcting the hypoxia and leaving the single ventricle responsible for supplying blood to the body only. To correct the PDA, ligation can be elected in this case. Since the hemi-Fontan and Fontan completion procedures are not carried out in the hospital, this poses a hindrance to the treatment of the patient.

## Conclusions

As discussed, symptomatic CCTGA is a rare finding at a young age of 15. The patient should be advised to report any episodes of light-headedness, unconsciousness, fatigue or dyspnea in order to warrant an investigation for congestive heart failure or any possible arrhythmia. Furthermore, such patients are at risk for endocarditis, thus proper prophylactic measures should be undertaken along with aggressive medical therapy. The patient, being a female, also needs to take special care during pregnancy. Association of marfanoid habitus with CCTGA is a rare clinical finding which demands further studies and in-depth research for revealing the underlying pathophysiology linking the two abnormalities.

## References

[1] Connelly MS, Liu PP, Williams WG, Webb GD, Robertson P, McLaughlin PR (1996). Congenitally corrected transposition of the great arteries in the adult: functional status and complications. J Am Coll Cardiol.

[2] Kilner PJ, Geva T, Kaemmerer H, Trindade PT, Schwitter J, Webb GD (2010 Apr 1). Recommendations for cardiovascular magnetic resonance in adults with congenital heart disease from the respective working groups of the European Society of Cardiology. Eur Heart J.

[3] Graf M, Zaczkiewicz M, Torzewski J, Zimmermann O (2012). Atrial fibrillation-induced cardiac shock: first manifestation of a congenitally corrected transposition of the great arteries in a 45-year-old man. Case Rep Cardiol.

[4] Hornung TS, Kilner PJ, Davlouros PA, Grothues F, Li W, Gatzoulis MA (2002). Excessive right ventricular hypertrophic response in adults with the mustard procedure for transposition of the great arteries. Am J Cardiol.

[5] Marelli AJ (2012). 69 - Congenital heart disease in adults. Goldman's Cecil Medicine, 24th ed.

[6] Mah K, Friedberg MK (2014 Sep). Congenitally corrected transposition of the great arteries: situs solitus or inversus. Circ Cardiovasc Imaging.

[7] Alghamdi AA, McCrindle BW, Van Arsdell GS (2006). Physiologic versus anatomic repair of congenitally corrected transposition of the great arteries: meta-analysis of individual patient data. Ann Thorac Surg.

[8] Warnes CA (2006). Transposition of the great arteries. Circulation.

[9] Graham TP Jr, Bernard YD, Mellen BG (2000). Long-term outcome in congenitally corrected transposition of the great arteries: a multi-institutional study. J Am Coll Cardiol.

[10] Wise-Faberowski L, Asija R, McElhinney DB (2019). Tetralogy of Fallot: everything you wanted to know but were afraid to ask. Paediatr Anaesth.

[11] Piran S, Bassett AS, Grewal J (2011 Jan). Patterns of cardiac and extracardiac anomalies in adults with tetralogy of fallot. Am Heart J.

[12] Zhang Y, Cai A, Sun W, Guo Y, Zhao Y (2011). Prenatal diagnosis of fetal congenitally corrected transposition of the great arteries. Prenat Diagn.

[13] Kutty S, Danford DA, Diller GP, Tutarel O (2018). Contemporary management and outcomes in congenitally corrected transposition of the great arteries. Heart.

[14] Prieto LR, Hordof AJ, Secic M, Rosenbaum MS, Gersony WM (1998). Progressive tricuspid valve disease in patients with congenitally corrected transposition of the great arteries. Circulation.

[15] Hornung TS, Bernard EJ, Jaeggi ET, Howman-Giles RB, Celermajer DS, Hawker RE (1998). Myocardial perfusion defects and associated systemic ventricular dysfunction in congenitally corrected transposition of the great arteries. Heart.

